# Compensatory Effect between Aortic Stiffening and Remodelling during Ageing

**DOI:** 10.1371/journal.pone.0139211

**Published:** 2015-10-01

**Authors:** Andrea Guala, Carlo Camporeale, Luca Ridolfi

**Affiliations:** DIATI, Politecnico di Torino, Torino, Italy; University of Giessen Lung Center, GERMANY

## Abstract

The arterial tree exhibits a complex spatio-temporal wave pattern, whose healthy behaviour depends on a subtle balance between mechanical and geometrical properties. Several clinical studies demonstrated that such a balance progressively breaks down during ageing, when the aorta stiffens and remodels by increasing its diameter. These two degenerative processes however, have different impacts on the arterial wave pattern. They both tend to compensate for each other, thus reducing the detrimental effect they would have had if they had arisen individually. This remarkable compensatory mechanism is investigated by a validated multi-scale model, with the aim to elucidate how aortic stiffening and remodelling quantitatively impact the complex interplay between forward and reflected backward waves in the arterial network. We focus on the aorta and on the pressure at the ventricular-aortic interface, which epidemiological studies demonstrate to play a key role in cardiovascular diseases.

## Introduction

The spatio-temporal pattern of pressure waves in the arterial tree is the result of complex fluid dynamic processes. Cyclic ventricular ejections generate forward waves that propagate, modify and interact with their backward travelling counterpart radiated from local and diffused reflection sites. The maintenance of physiological conditions depends on a subtle balance between geometrical and mechanical characteristics of the arterial tree. In healthy young subjects, backward waves are small, largely damped and return to the heart just after the end of ejection, lowering the left-ventricular work and enhancing coronary perfusion. In contrast, in pathological conditions this balance breaks down and higher and faster waves occur in the arterial system.

An emblematic example of pressure pattern worsening happens during ageing, when arterial tissues underneath a degenerative process which mainly originates from the cycling stress state and a number of pro-inflammatory mechanisms [1, 2]. The consequence is a plastic remodelling (i.e., an increment of vessel diameters) and a general stiffening of the large arteries. Such structural changes have a strong impact on the generation, propagation and reflection of pressure waves in the arterial tree, resulting in an increased aortic systolic pressure and left-ventricular work [3]. Since these are key determinants of heart failure [4], population studies confirm the strong association between age and cardiovascular morbidity and mortality [5]. In fact, although 90% of cardiovascular diseases are estimated to be preventable, they are still the leading cause of death in the world.

In the last years, several clinical studies [5–8] explored the age-induced fluid dynamic processes which are responsible for the decline of the arterial tree efficiency. Differently, the modelling approach has been adopted rarely, although mathematical models have contributed to understand other features of the cardiovascular system (e.g. [9–11]). The seminal work by Liang *et al* [12] used a multi-scale model to study the hemodynamics of an ageing arterial system. They investigated several features (impact of heart ageing, age-induced wave pattern modifications, etc.) and mentioned the compensatory role of the arterial stiffening and remodelling. The aim of the present work is to provide a thorough analysis of this important balance between two degenerative processes, whose effects on the arterial fluid dynamics tend to compensate for each other, reducing the negative effects on arterial pressure pattern they would have had if they acted individually. Such a balance in fact plays a key role in maintaining pressure close to the physiological values, in spite of the occurrence of degenerative processes.

We will focus on the aortic region and central pressure, namely the pressure at the ventricular-aortic interface (aortic root). Central pressure plays a key role in predicting cardiovascular failure [13] and its alterations have a high prognostic value. Our study is based on a validated multi-scale mathematical model [14] that allows us to analyse the spatio-temporal wave pattern and to elucidate the interplay between forward and backward waves. On the basis of literature data, the age-dependent characterization of a number of model parameters is here introduced with the aim to investigate the hemodynamic modifications during ageing.

## Methods

### Cardiovascular model

Let us consider an axisymmetric elastic vessel of radius *R*(*x*, *t*) whose walls are impermeable, tapered, longitudinally-tethered, and are subjected to small and radial deformations. The basic assumptions are: (i) laminar flow; (ii) no pressure variation over the cross section; (iii) Newtonian incompressible blood, characterized by constant density, *ρ*, and kinematic viscosity, *ν*; (iv) no gravity effects (i.e., the subject is supine).

After integration over the cross section, mass and momentum balance equations in cylindrical coordinates read

$$\begin{array}{c} \frac{\partial A}{\partial t}+\frac{\partial Q}{\partial x}=0,\;\frac{\partial Q}{\partial t}+\frac{\partial }{\partial x}(\beta \frac{Q^{2}}{A})=-\frac{A}{\rho }\frac{\partial P}{\partial x}+F, \end{array}$$ (1*a*, *b*)

where *x* is the longitudinal coordinate, *t* is time, *P*(*x*, *t*) is the pressure, *A*(*x*, *t*) is the vessel cross-section, $Q(x,t)=\int_{0}^{R}u(r,x,t)rdr$ is the flow rate (the longitudinal velocity *u* being also a function of the radial coordinate *r*), $\beta =2\pi AQ^{-2}\int_{0}^{R}u^{2}(r,x,t)rdr$ is the so-called Coriolis coefficient and $F=2\pi R\nu {\left[\frac{\partial u}{\partial r}\right]}_{r=R}$ is the the wall friction per unit length.

The terms *β* and *F* in Eq (1) require a characterization of the velocity profile. Several shapes have been proposed in the literature such as flat profiles [15], parabolic profiles [16] and profiles joining a linear viscous layer and a flat inviscid core [17]. We choose here a compromise between physiological likelihood and computational complexity imposing a central flat profile joined to a parabolic boundary layer of fixed thickness *δ*. Lighthill [18] suggested that *δ* remains almost constant in large arteries becoming comparable to the arterial radius in small vessels. Its rough estimate is given from the equilibrium between inertial and viscous forces, $\tilde{\delta }\;=\;\sqrt{\nu T/2\pi }\approx$ 1 mm, where *T* is the cardiac period. The velocity therefore reads *u*(*r*, *x*, *t*) = G(*r*)*u*(0, *x*, *t*) where G = (*R* − *r*) / (2*Rδ* − *δ* ^2^) in the boundary layer (i.e., *r* > *R* − *δ*) and G = 1 in the core. Notice that $\delta =\tilde{\delta }$ if $R\;>\;\tilde{\delta }$, while *δ* = *R* otherwise. By using the definition of the flow rate *Q*, one finally obtains

$$\begin{array}{c} \beta =\frac{4}{3}\frac{3A^{2}-4A^{3/2}\sqrt{\pi }\delta +2A\pi \delta^{2}}{{\left(2A\;-\;2\sqrt{A}\sqrt{\pi }\delta +\pi \delta^{2}\right)}^{2}},\;F\;=\;\frac{8AQ\sqrt{\pi }\nu }{\delta (6A\sqrt{\pi }\delta -4\sqrt{A}\pi \delta^{2}\;+\;\pi^{3/2}\delta^{3}-4A^{3/2})}. \end{array}$$ (2*a*, *b*)

Eq (1) still require a constitutive closure linking pressure and cross area. Aiming to reproduce the arterial non-linear viscoelastic behaviour [19], one can pose $P=\sum_{i=1}^{4}B_{i}A^{i-1}\;-\;B_{5}A^{-1/2}\text{d}Q/\text{d}x$, where the functions *B*_*i*_ characterize the stiffness of the vessel wall as a function of wave celerity, so-called pulse wave velocity *c*. Their derivation is provided in Guala *et al* [14].

According to the above framework the *k*-th vessel of the arterial tree is described by the quantities *Q*_*k*_(*x*, *t*) and *A*_*k*_(*x*, *t*), where *x* ranges between zero and the vessel length *l*_*k*_. Diameters, tapering rates and topology of the arterial network (48 branches) as well as distal model parameters are provided in Reymond *et al* [10]. Suitable boundary conditions must be imposed (dot refers to time derivative):

1. at the aortic root (*k* = 1, *x* = 0)
  $$\begin{array}{c} \begin{array}{c} L{\dot{Q}}_{1}+\sigma_{0}Q_{1}+\sigma_{1}\left|Q_{1}\right|Q_{1}=\sigma_{2}{\left(1-\cos\theta \right)}^{4}\left(P_{v}-P_{1}\right),\\ I\ddot{\theta }+\kappa_{f}\dot{\theta }=\kappa_{a}Q_{1}\cos\theta +\kappa_{p}\left(P_{v}-P_{1}\right)+\kappa_{v}Q_{1}𝓗\left[P_{v}-P_{1}\right]\sin\theta ,\\ \dot{V}=\left(P_{a}-P_{v}\right)/R_{m}-Q_{1}, \end{array} \end{array}$$ (3*a*–*c*)
2. at each bifurcation between a generic *k*_*f*_ (father) vessel and two *k*_*d*1_ and *k*_*d*2_ (daughter) vessels
  $$\begin{array}{c} {\frac{P_{k_{f}}}{\rho }|}_{x=l_{k_{f}}}\;\;+\;\frac{{\left[Q_{k_{d1}}\;+\;Q_{k_{d2}}\right]}_{x=0}^{2}}{2A_{k_{f},x=l_{k_{f}}}^{2}}\;=\;{\left[\frac{P_{k_{d1}}}{\rho }\;+\;\frac{Q_{k_{d1}}^{2}}{2A_{k_{d1}}^{2}}\right]}_{x=0}=\;{\left[\frac{P_{k_{d2}}}{\rho }\;+\;\frac{Q_{k_{d2}}^{2}}{2A_{k_{d2}}^{2}}\right]}_{x=0}, \end{array}$$ (4)
3. at *N* distal sites (*x* = *l*_*k*_*n*__, *n* = *n*_1_…*n*_*N*_)
  $$\begin{array}{c} r_{2}C\left(r_{1}{\dot{Q}}_{k_{n}}-{\dot{P}}_{k_{n}}\right)=\left(P_{k_{n}}\;-\;P_{ven}\right)-\left(r_{2}\;+\;r_{1}\right)Q_{k_{n}}. \end{array}$$ (5)

Following [20], Eq (3*a*) is the pressure-flow law for the aortic valve, where *L* accounts for the inertance of the fluid, *σ*_0_ is the viscous resistance, *σ*_1_ is the turbulent flow separation coefficient and *θ*(*t*) is the opening angle of aortic valve. Eq (3*b*) is the angular momentum balance for the aortic valve, where *I* is the rotational inertia of the aortic leaflet and the coefficients {*κ*_*f*_, *κ*_*a*_, *κ*_*p*_, *κ*_*v*_} account for the frictional resistance, the dynamic and the static forces on the leaflets, and for the action of the vortex downstream to the valve, respectively, being H[*P*_*v*_ − *P*_1_] = 1 if *P*_*v*_ > *P*_1_ and zero otherwise [9]. Eq (3*c*) imposes the mass conservation for the ventricular filling, *V*(*t*), where the first term in the right-hand side accounts for the mitral flow as an ideal diode, with the resistance *R*_*m*_ switching to infinite for negative pressure difference. *P*_*a*_ is the left atrium pressure, that is set to a constant value, for simplicity. The left-ventricular pressure follows the relation *P*_*v*_ = (*V* − *V*_0_)*E*(*t*), wherein the force of contraction is modelled through the elastance function. *E*(*t*) = *E*_*m*_(1 − Φ) + Φ*E*_*M*_; Φ(*t*/*t*_*s*_) is an activation function that accounts for a particular waveform during systole and depends on the systolic duration *t*_*s*_, while *E*_*m*_ and *E*_*M*_ are the minimum and the maximum values of elastance. The left-atrium pressure is instead set to a constant value *P*_*a*_. Coefficient values are provided in Korakianitis *et al* [9].

The internal boundary conditions (4) are imposed at the bifurcations in order to assure the continuity of total pressure and the mass conservation Finally, a set of distal boundary conditions (5) are imposed at the terminal sections of the *N* distal vessels, through three-element Windkessel models. They are lumped representations of microcirculation volumes by means of a resistance *r*_1_, followed by a parallel combination of resistance *r*_2_ and capacitance *C* [19]. *P*_*ven*_ = 5 mmHg is a constant pressure corresponding to the microcirculation and the venus system, which is therefore not dynamically modelled.

The full set of hyperbolic partial differential Eqs (1)–(5) is solved by a Runge-Kutta Discontinuous-Galerkin method flanked by numerically efficient compatibility conditions, written in terms of pseudo-characteristic variables that guarantee well-posedness of the problem [further numerical details are reported by Guala *et al* [14]].

### Age-dependent modelling

With the aim of reproducing the ageing of the heart-arterial system, we have considered several physiological trends reported in the literature. It is widely accepted that the aorta and common carotids dilate, thicken and non-uniformly elongate with ageing [7, 21–23], while the behaviour of medium vessels is still controversial [24]. In this respect, since the majority of literature does not find any significant correlation [25, 26], we choose to keep the geometrical characteristics of the muscular arteries unchanged with age.

The aorta is subdivided into four different segments and the temporal growth rate of diameters *D*_*k*_, thickness *h*_*k*_ and lengths *l*_*k*_ is given by $\dot{\xi }$ = *α*_*ξ*_, where *ξ* = {*D*, *h*, *l*}. All coefficients *α*_*ξ*_ have been obtained by linearly fitting the data proposed by Virmani *et al* [21] and are reported in Table 1 (age is given in years, lengths in mm and velocity in m/s). Henceforth, this component of ageing will be referred to as remodelling.

**Table 1:** Values of the age-dependent coefficients. V91: [21]; S08: [34]; V10: [32]; H10: [7].

	Ascending	Thoracic	Suprarenal	Subrenal	Carotid	ref.
*α*_*D*_ [*y* ^−1^]	0.1282	0.1467	0.1425	0.1038	0.0239	V91
*α*_*h*_ [*y* ^−1^]	0.0041	0.0071	0.0064	0.0088	0.007	V91
*α*_*l*_ [*y* ^−1^]	0.9	0	0	0	0	S08
*α*_*C*1_ [*y* ^−1^]	-0.004	0.011	0.005	0.06	-0.118	V10
*α*_*C*2_⋅10^−3^ [*y* ^−2^]	0.44	0.58	0.53	0.29	1.94	H10

In addition, the aorta stiffens with age [2, 3, 6, 7, 22–24, 27, 28] in a non-homogeneous way [7]. Differently, the stiffening of other, small and muscular arteries is still matter of debate. Even though a pulse wave velocity growing trend has been found in both upper and lower limb arteries [29], no age-related changes on femoral [26, 30], iliac [27] and carotid-brachial [31] mean celerities have been recently measured. Therefore, we will only consider the growth of the pulse wave velocity of aorta and carotid arteries. The stiffening trend is deduced by exploiting both the large data set of carotid-femoral pulse wave velocities reported by Vermeersch *et al*. [32] and the location-specific trend proposed by Hickson *et al* [7]. In particular, these latter data provide the stiffening rates in different vessel segments, which are imposed to give the overall carotid-femoral pulse wave velocity growth found by the former. The stiffening trend is then obtained as $\dot{c}$ = *α*_*c*1_ + 2*α*_*c*2_(*a* − *a*_0_), where *a* is the age and coefficients are reported in Table 1. For both remodelling and stiffening, the offset *a*_0_ = 20 years refers to the complete absence of ageing.

The dramatic changes affecting large arteries have a counterpart on the left-ventricle, which strengthens in order to preserve the amount of blood ejected [12]. In this respect, we follow Redfield *et al* [6] by imposing ${\dot{E}}_{M}$ = 0.005*E*_*M*_. Finally, even though the heart rate is unchanged with age, several studies have reported a growth in systole duration so that, following Mangoni *et al*. [33], we set *t*_*s*_ = 0.37 + 0.372 ⋅ 10^−3^ *a* (s).

It is worth noticing that, since the modelling of ageing adopted in this work is based on large data sets, the following results refer to representative average conditions and subject-specific particular cases are not considered here.

### Waveform decomposition

Waveforms can be decomposed into forward and backward propagating components. The latter are due to local reflections at mechanical or geometrical discontinuities (e.g., arterial bifurcations) and distributed reflections induced by vessel tapering. Since friction is negligible in the aorta [19], one can write

$$\begin{array}{c} P_{f}=\frac{P+Z_{C}Q}{2}\;P_{b}=\frac{P-Z_{C}Q}{2}, \end{array}$$ (6)

where subscripts *f* and *b* refer to forward and backward waves, respectively, and *Z*_*C*_ = *ρc*/*A* is the characteristic impedance. This is a local property of an artery, corresponds to the high frequency limit of the impedance spectrum, and quantifies the pressure-flow relation in absence of reflection, when pressure and flow are in phase [19, 35].

### Reflection coefficients

Every time a pressure wave encounters a change in mechanical or geometrical properties of the vessel, a part of the forward pressure wave is reflected and backward propagating waves are generated. Although diffused reflections arise everywhere, bifurcations are the main source of reflections. In this regard, the bifurcation reflection coefficient can be assessed as

$$\begin{array}{c} P_{b}=\Gamma \cdot P_{f}\;\text{with}\;\Gamma =\frac{Z_{C(in)}-\sum Z_{C(out)}}{Z_{C(in)}+\sum Z_{C(out)}} \end{array}$$ (7)

where Γ is the bifurcation-specific reflection coefficient and subscripts *in* and *out* refer to bifurcation inlet and the outlet sections, respectively [36]. A positive (negative) reflection coefficient means that part of the forward pressure wave is reflected as a compression (expansion) wave, so that the backward pressure wave adds (subtracts) to the incident wave.

Focusing on the aorta, a bifurcation can be traversed in two ways: following either forward waves (i.e., downstream) or backward waves (upstream). Accordingly, two reflection coefficients exist for each bifurcation and both of them are affected by ageing.

## Results and Discussion

**Fig 1:**
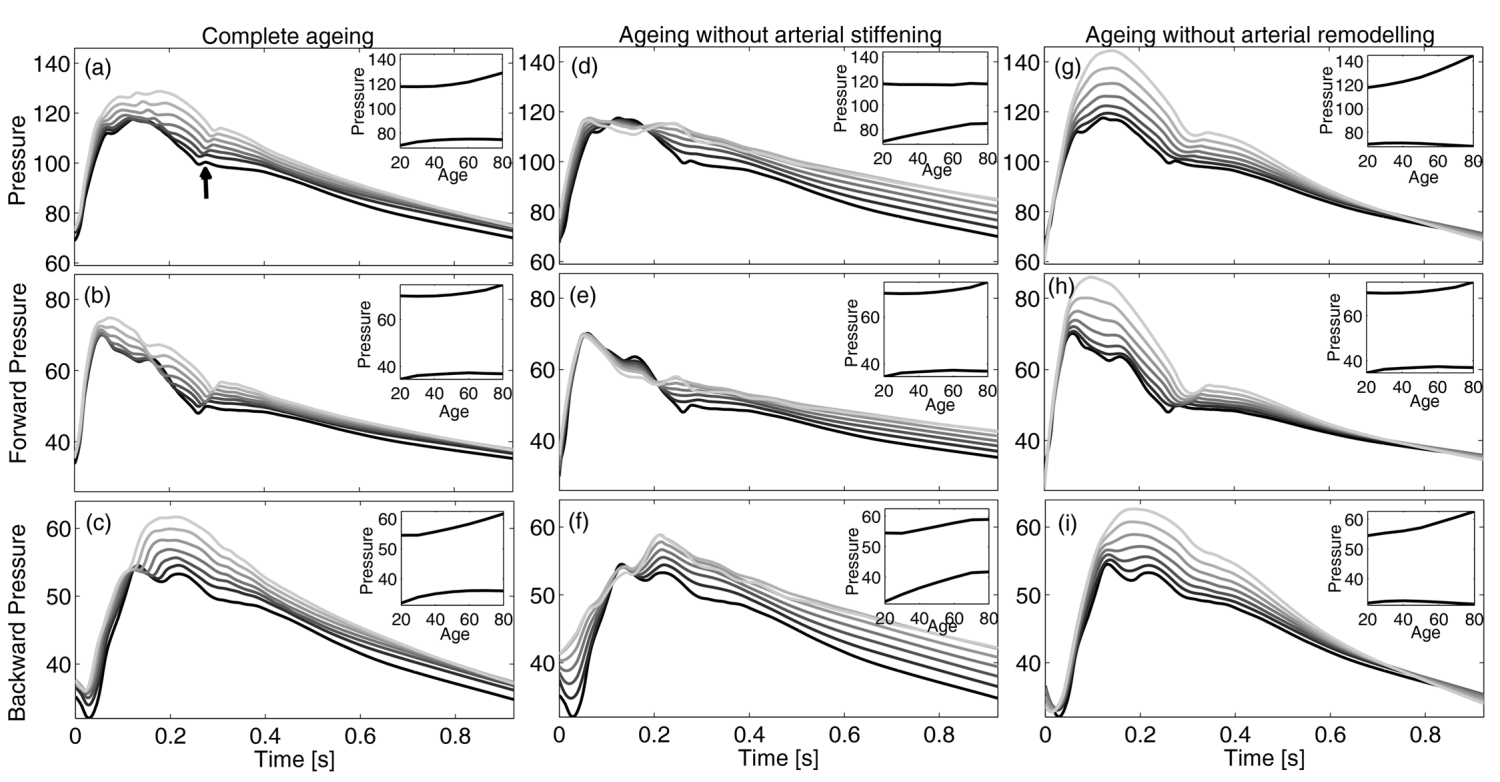
Pressure waves at ascending aortic section. Upper row: total pressure, *P*, with physiological ageing (a), when the pulse wave velocity increase is removed (d), or the plastic geometric remodelling is not accounted (g). Middle and lower rows report the corresponding forward and backward pressures, respectively. The darker the younger, from 20 to 80 years old. Insets show the evolution of maximum and minimum pressures during ageing.

Fig 1a shows the evolution of central pressure behaviour during ageing. At a young age, systolic portion exhibits a clear inflection between early and late systolic peaks, while diastolic decay has a weak local bump just after the dicrotic notch (marked with an arrow in Fig 1a). With age, a second late systolic peak arises, which tends to join with the first peak and overwhelms it, while inflection disappears. At the same time, diastolic decay becomes more and more regular and concave.

Our model shows that, during ageing, the systolic value grows with increasing rate, resulting in an increment of more than 10 mmHg from 20 to 80 years old. This is in agreement with the 12 mmHg increase of the systolic pressure invasively measured by McEniery *et al* [22] in a similar age range. Some works [7, 37] report much higher values, but they used non-invasively calibrated transfer functions and the systematic error due to this practice explains such differences [38]. On the other hand, diastolic pressure firstly grows until around age 55 and then slightly decreases with advancing age. Both the increasing and decreasing pattern behaviour, as well as the position of the maximum around 55 years, are well documented in literature [22, 23, 37]. Systolic and diastolic patterns determine that pulse pressure, i.e. the difference between maximum and minimum pressure, firstly decreases and then inflates [22, 23]. More specifically, at age 20 the pulse pressure is 48 mmHg, then it declines to its minimum value of 44, which is reached around age 42. Afterwards, pulse pressure increases and reaches a value of 54 mmHg at age 80. Very similar values have been obtained by McEniery *et al* [22], who measured values of 50, 47 and 57 mmHg at 20, 40 and 80 years old, respectively.

Moreover, the reduction of the distance between the foot of the aortic pressure wave and the inflection point—i.e., the reflected wave transit time (RWTT)—is visible in Fig 1a. This quantity is systematically reduced during ageing, with values RWTT = {0.131, 0.126, 0.122, 0.112} at ages 20, 40, 60 and 80, respectively. Very similar values have been observed [31, 39]. Another prognostic indicator of the evolution of the aortic pressure wave is the augmentation index (Aix). In the same age sequence, the model predicts Aix = {3,4,12,17}, which is in good agreement with the clinical data by McEniery *et al* [22], although they are slightly lower in the middle-age range.

Panels 1*b* and 1*c* show forward and backward central pressure waves, respectively. The forward waves grow steeply in the early systolic phase, attain their maximum value when total pressure *P* presents the local inflection, and then start to decline, although other (smaller) peaks occur during decay when backward waves are reflected at the ventricular-aortic interface. Backward component occurs with a delay and its maximum is aligned with the maximum of total pressure.

During early ageing, small changes affect forward pressure component, as maximum and minimum values are substantially conserved (though with a slight delay). This feature is due to the well-balanced interaction among (i) the increased force of heart contraction, which tends to enhance maximum values, (ii) the aortic root characteristic impedance, which decreases until around age 60 and then increases (inducing a similar trend to forward component), and (iii) the increased amount of backward pressure waves re-reflected at the ventricular-aortic interface, which enhances the second peak of the forward component. Until about 60 years of age, the effect of the increased force of contraction is smoothed out by the reduction of characteristic impedance (as found by Segers *et al* [40]), while after this age the characteristic impedance increases, leading to an increase of the forward peak.

On the contrary, backward component (panel 1*c*) exhibits more significant modifications: the second peak occurs earlier as a symptom of the growing wave celerity and becomes larger, overwhelming the first one and dominating total pressure evolution. In contrast, minimum values are only slightly modified during ageing. Such age-induced behaviours of both forward and reflected pressure waves are coherent with clinical findings [8].

The central and right columns of Fig 1 show the effects of arterial stiffening and remodelling, respectively, on the central pressure waveform. These results correspond to two sets of runs where wave celerity or vessel diameters were maintained equal to the values corresponding to a young subject. Panels *d* and *g* show how arterial stiffness and remodelling play antagonistic roles in the central pressure pattern: with age, if there are no changes in aortic celerity, systolic pressure is preserved, while diastolic pressure grows considerably (panel *d*); on the contrary, if vessel diameters are maintained (panel *g*), there is a dramatic increase of systolic pressure and preservation of diastolic values occur during ageing. A similar scenario has been observed in a longitudinal study [41], where a coupled analysis of sex differences in the aortic dilation and in the aortic stiffening allowed the opposite effect of stiffening and remodelling on the pulse pressure to be highlighted. The direct (inverse) dependence of the characteristic impedance *Z*_*C*_ on wave celerity (vessel area) partially explains this behaviour: when stiffening is inhibited, *Z*_*C*_ decreases due to remodelling leading to a weak decrease of forward component (panel *e*); differently, when remodelling is absent *Z*_*C*_ strongly grows and the systolic portion of the forward wave increases accordingly (panel *h*).

The suppression of stiffening contributes also to the delay of the second pressure peak, which is related to the backward wave, and to its reduced growth with respect to normal ageing (panel *f*). Indeed, when the celerity is conserved, the RWTT does not change substantially and the backward travelling wave peak maintains its location with age, thus contributing to the rise of diastolic rather than systolic pressure even in the elderly. On the contrary, the remarkable enhancement of forward wave induced by the lack of remodelling contributes to the dramatic increment of backward wave in the systolic segment (panel *i*), whose age-induced anticipation is preserved.

In order to understand how the backward wave pattern evolves with ageing, it is useful to focus on vessel bifurcations. In fact, these are the main sources of backward components in the aorta, while vessel tapering only has a minor effect.

Relation (7) makes clear that ageing-induced changes of the characteristic impedances have a twofold effect on backward waves. Firstly, variations in the incident waves (panels 1*b*, *e*, *h*) induce alterations of the reflected waves. Secondly, an unbalanced rise of characteristic impedances increases the bifurcation mismatch. The backward wave pattern shown in panels *c*, *f*, *i* is essentially the result of the interplay of these two actions.

**Fig 2:**
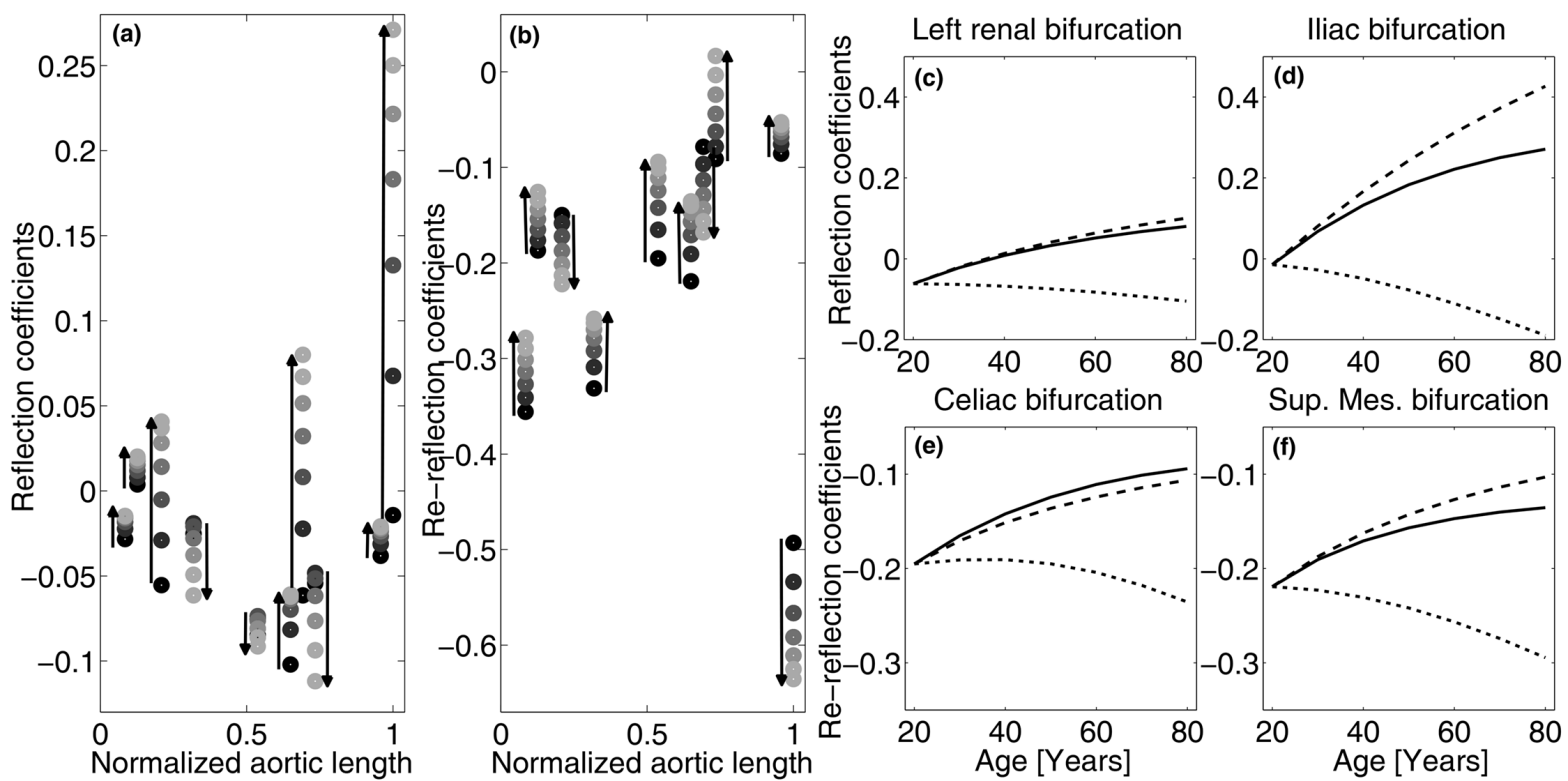
(a,b) Ageing-induced changes of reflection coefficients at main aortic bifurcations as seen by forward (a) and backward (b) waves travelling along the aorta. From left: brachiocephalic, left carotid, left subclavian, intercostals, celiac, superior mesenteric, left and right renal, and inferior mesenteric bifurcations. Circles indicate reflection coefficient values from 20 to 80 years of age with 10 years step, the darker the younger. (c-f) Reflection coefficients at left renal (c) and iliac (d) bifurcations, and re-reflection coefficients at celiac (e) and superior mesenteric (f) bifurcations during normal ageing (solid line), without aortic stiffening (dashed line), and without remodelling (dotted line).

Fig 2a and 2b shows the alterations of forward and backward reflection coefficients for the main aortic bifurcations. In young subjects, reflection coefficients for forward waves are generally close to zero, confirming that the aorta is well-matched for forward waves [36, 42]. During ageing, low values of Γ are substantially preserved in all locations, even though iliac bifurcation exhibits a sharp increment and subclavian and renal coefficients become positive. Again, these modelling findings are in agreement with clinical data [42].

Consequently, with advancing age the intensity of backward wave generated at the iliac increases significantly and backward compression in place of expansion waves are generated at subclavian and renal bifurcations, resulting in an increase in reflected waves, as shown in Fig 1c and observed in several clinical studies [8, 11, 40].

Backward waves generated at a bifurcation are partially re-reflected at the upstream bifurcations. As shown in Fig 2b, the reflection coefficients for backward waves are all negative and their modulus is generally higher than the corresponding forward ones. Particularly at a young age, bifurcations are not as well-matched for backward propagating waves as they are for their forward counterpart [36], entailing a protective mechanism called *wave trapping* [11] which substantially smooths out backward waves before they reach the heart. During ageing, most of the backward reflection coefficients move towards zero resulting in an increase in the amount of backward waves able to pass through bifurcations. Namely, the healthy filter action on the backward waves due to the (upstream) impedance mismatch decreases progressively with age. This fact, combined with the increase of forward waves, explains the wave pattern shown in Fig 1c.

In order to disentangle the roles played by aortic stiffening and remodelling, panels 2*c*–*f* show their different contributions to some key reflection coefficients. Remodelling is the most impacting factor and has a negative effect on the capability of the aortic bifurcation system to prevent excessive backward pressure waves. In fact, diameter remodelling increases the forward coefficients (see panels *c* and *d*), promoting the occurrence of high backward waves, and reduces the backward coefficients (panels *e* and *f*) and consequently the wave trapping effect. On the contrary, stiffening tends to mitigate these negative impacts, demonstrating its compensatory role with respect to remodelling. This is a key point, because stiffening is generally considered to have only negative effects on the arterial pressure pattern. Instead, reflection coefficients show that stiffening gives a (small) positive contribution, as it reduces the difference between central and peripheral pulse wave velocities [31].

**Fig 3:**
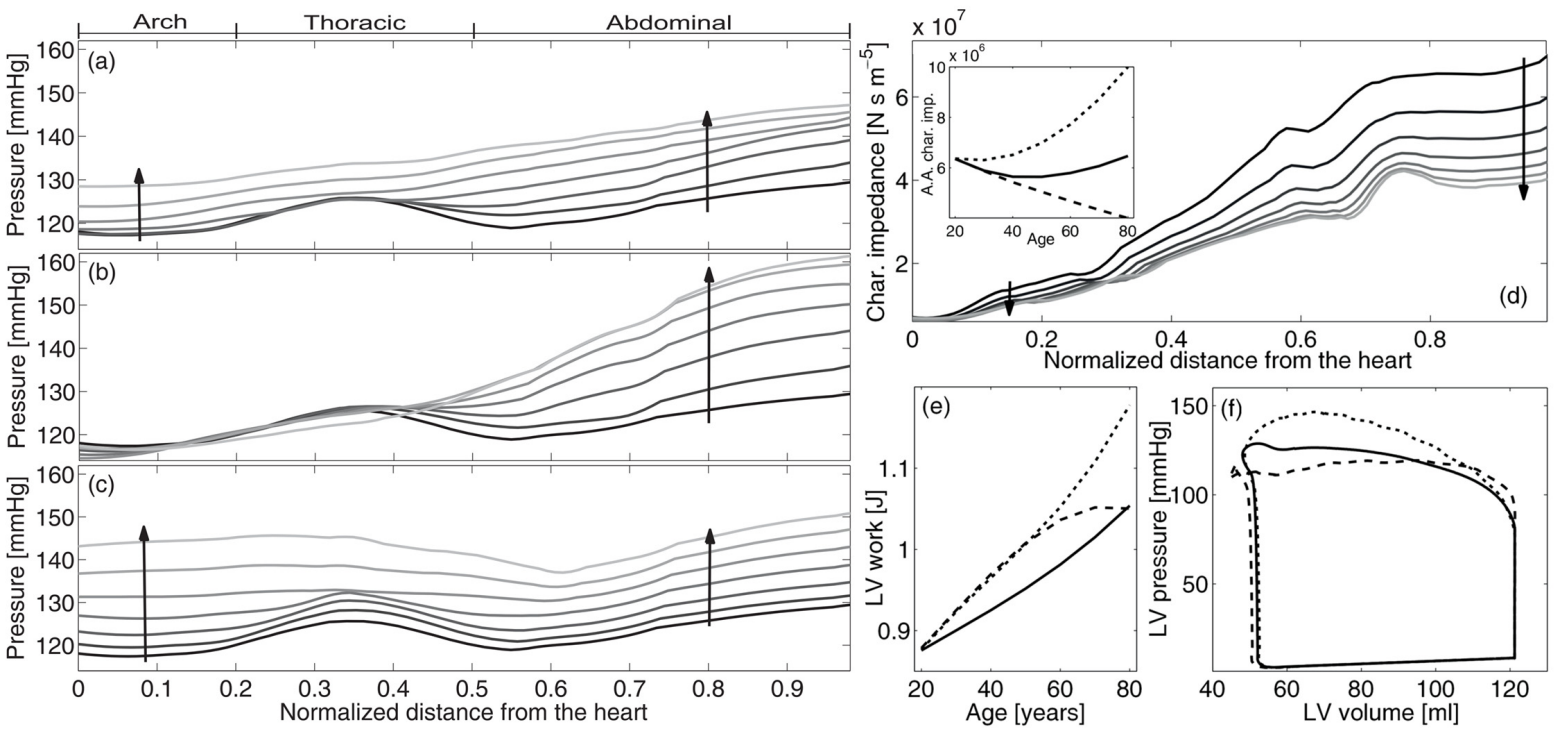
Left: Systolic pressure along the aorta at different ages. From top to down: (a) physiologic ageing, (b) ageing without increase of pulse wave velocity and (c) ageing without plastic geometric remodelling. Distances are normalized to aortic length. From 20 to 80 years of age, with 10 years step. The darker the younger (see arrows). Right: characteristic impedance at different ages along the aorta (the inset shows aortic root values with age)(d), LV work during ageing (e) and LV pressure-volume loops at 80 years old (f) for complete ageing (continuous line) and in the absence of arterial stiffening (dashed line) and remodelling (dotted line).

Fig 3 focuses on the effects of ageing along the aorta. As already shown by Pedley [35], systolic pressure increases downstream, but key features become apparent when ageing is properly modelled. Coherently with in-vivo observations [43–45], simulations highlight a local peak at the thoracic aorta for young people (see Fig 3a). This aspect has so far been unexplored and has two causes. Firstly, aorta tapering entails an increment of the characteristic impedance moving away from the heart: in fact, Fig 3d shows an eight-fold increase of *Z*_*C*_ from the root to the abdominal section, especially marked at thoracic sections [43]. A large amount of diffused reflections are thus generated at the thoracic aorta, and add to forward waves enhancing the amplification just upstream from this region. Secondly, backward pressure waves from the iliac bifurcation encounter a progressive reduction of characteristic impedance at the thoracic segment, which generates diffuse negative re-reflections that propagate downstream, reducing pressure after the peak. This second cause is mirrored for forward waves in the negative reflection coefficient of the renal bifurcations (Fig 2a–2c). In summary, negative reflected waves are subtracted from forward waves after the thoracic section, contributing to the local minimum.

During ageing, the overall growth in the systolic pressure along the aorta is mainly due to the rise in its aortic root value. The similar growth of proximal and distal sections entails a reduction of pulse pressure amplification with age, as found in several clinical studies [22, 24, 31, 45–47]. Moreover, the increase of characteristic impedance along the aorta is dampened with age [48]: from 20 to 80 years of age, abdominal values of *Z*_*C*_ change from being 8 to 5 times the characteristic impedance at the aortic root and, in particular, in the thoracic aorta the increase reduces from +150% to +80% in the same age range (see panel 3*d*). It follows that the spatial gradient of systolic pressure upstream the thoracic aorta is reduced, contributing (from upstream) to smooth away the peak. From downstream, the peak tends to disappear due to the age-induced changes of reflection coefficients. The growth of the reflection coefficient at renal and iliac bifurcations (notice that the renal coefficients become positive) and the tendency towards zeros of the re-reflection coefficients entails an increase in backward waves that contribute to the general trend of pressure.

If stiffening is removed from ageing (Fig 3b), the consequent dramatic decay in the characteristic impedance (see inset in Fig 3d) inhibits the systolic pressure in the first portion of the aorta, while both the increase in the positive reflections at renal and iliac bifurcations (Fig 2c and 2d) and the decrease in re-reflections (especially the iliac one) induce a remarkable growth of pressure in the abdominal aorta. A negative correlation between pulse pressure amplification and pulse wave velocity has been observed in clinical studies [31, 45–47] and demonstrated through an approximated analytical theory [49]. Differently, Fig 3c shows that if remodelling is removed, systolic pressure grows in the ascending aorta more than in the abdominal sections. The reason is the increase of aortic root characteristic impedance and the decrease of renal and iliac bifurcation reflection coefficients (Fig 2c and 2d), respectively.

Systolic pressure behaviour along the aorta confirms the antagonistic role played by the age-induced increase of pulse wave velocity and diameters: the former enhances aortic root pressure and reduces abdominal values, while the latter lessens proximal more than distal pressure.

The changes affecting large arteries have a counterpart on the work of left ventricle. The left ventricle strengthening with age guarantees the conservation of cardiac output against the increased afterload, as reproduced by the present model (data not shown) and observed in clinical studies [12]. Along with the conservation of heart rate, this means that the model-predicted mean flow ejected by the left ventricle does not change with age. Being the leakage through the arterial walls not considered in the present model, it follows the conservation of mean flow throughout the arterial network. Moreover, the increase of ejection time produces a slight reduction of the ascending aortic flow peak with age. Finally, peripheral sections (e.g., the radial, brachial, femoral, and tibial sections) are characterized by a slight steepening of the flow wave with advancing ageing, which results in a moderate growth of the flow peak.

In Fig 3e the left-ventricular work at different ages is shown for regular ageing and in the absence of aortic stiffening or remodelling. As expected, left-ventricular work physiologically grows with age [50], although less than in both artificial cases, underlying the importance of the stiffening-remodelling compensation. In particular, removing arterial stiffening reduces the late systolic ventricular pressure but raises early values and enhances cardiac output, as shown in Fig 3f. Until about middle age, the resulting left-ventricular work is dominated by the concomitant increase of early systolic pressure and cardiac output, thus growing more than the physiological case. On the other hand, in elderly people the strong reduction in late systolic pressure values lowers the growing trend, finally leading to the physiological left-ventricular work values. Conversely, the absence of arterial enlargement augments late systolic pressure but conserves early values and slightly reduce cardiac output. The corresponding left-ventricular work grows sharply, being dominated by the late systolic pressure values.

## Conclusions

In recent years, fluid mechanics has proved to be increasingly important in uncovering both physiological and pathological processes of the cardiovascular system. The present work confirms the goodness of this approach. The detrimental increase of maximum pressure and left ventricular work during ageing in fact results from the impairment of the subtle fluid dynamic balance between generation and propagation of forward waves and reflections and damping of backward waves. Our quantitative outcomes shed light on how ageing-induced aortic stiffening and remodelling affect this balance: (i) the former enhances the first pressure pulse generated at the ventricular-aortic interface during ejection, while the latter damps it; (ii) although stiffening tends to decrease reflection coefficients at network bifurcations, their remodelling-induced large growth prevails, increasing the total amount of reflection; (iii) aortic remodelling undermines the protective wave-trapping mechanism on reflected pressure waves while stiffening enhances it; (iv) aortic stiffening and remodelling exhibit a compensatory effect on the pulse pressure amplification: the former reduces it, while the latter augments it; (v) by contrast, both stiffening and remodelling contribute to limit the growth of left-ventricle work with age.

These results suggest that an excessive imbalance between aortic stiffness and geometric remodelling during clinical treatment of elderly subjects should be avoided. As arterial geometrical adaptation develops over long time-scales, a fast drug-induced large reduction of pulse wave velocity can be dangerous [47]. A dramatic reduction of the pulse wave velocity in a dilated aorta can lead to an increase of the left ventricular work and pulse pressure amplification, with potential detrimental effects on organs such as the kidneys.
